# An Insight into the Growing Concerns of Styrene Monomer and Poly(Styrene) Fragment Migration into Food and Drink Simulants from Poly(Styrene) Packaging

**DOI:** 10.3390/foods10051136

**Published:** 2021-05-20

**Authors:** Asmaa Ajaj, Shayma J’Bari, Anthonia Ononogbo, Federico Buonocore, Joseph C. Bear, Andrew G. Mayes, Huda Morgan

**Affiliations:** 1 School of Life Sciences, Pharmacy and Chemistry, Kingston upon Thames, London KT1 2EE, UK; a.ajaj@kingston.ac.uk (A.A.); k1303038@kingston.ac.uk (S.J.); a.ononogbo@kingston.ac.uk (A.O.); f.buonocore@kingston.ac.uk (F.B.); j.bear@kingston.ac.uk (J.C.B.); 2 School of Chemistry, University of East Anglia, Norwich Research Park, Norwich, Norfolk NR4 7TJ, UK; Andrew.Mayes@uea.ac.uk

**Keywords:** food contact materials, styrene, migration, poly(styrene) fragments, food simulants, oligomer clusters, microplastics

## Abstract

Poly(styrene) (PS) has been heavily utilised in disposable food packaging due to its insulating properties, optical translucency, and long-shelf life. Despite these desirable characteristics, (PS) poses toxicity concerns to human’s health through styrene monomer leaching into foodstuffs. Environmental and marine hazards are another growing concerns due to improper and/or absence of recycling strategies and facilities. This preliminary work aims to investigate the effect of temperature, food composition and contact times on the migration of the styrene monomer from poly(styrene) food contact materials into food simulants. Poly(styrene) cups showed a relatively low level of styrene migration with the highest being 0.110 µg/mL, whereas food containers showed a much higher level of styrene leaching with up to 6 µg/mL. This could be due to an increase in the hydrophobicity of the simulants’ characteristics from low to high fat content and the increase in the testing temperatures from 5 °C to 70 °C. ANOVA statistical analysis is used to compare the means of three or more groups of data, whereas t-test analysis is used to compare means of two groups. This was carried out on each individual sample to determine the significance of changing the temperature, simulant type, or both on the level of migration observed in the results. All significant values were tested at 95% confidence level *p <* 0.05, concluding that fat content and high temperatures were found to significantly increase the level of styrene migration. Nile Red staining method was used to demonstrate that particulate poly(styrene), as well as styrene monomer, migrated into tested food simulants from typical containers, which is becoming a cause for concern as evidence of microplastic ingestion increases.

## 1. Introduction

Plastics are synthetic polymeric structures consisting of repeating units of monomers, produced through addition or condensation polymerisation reactions [1]. These are built up to form large macro-molecular chains held together by inter-molecular and intra-molecular interactions resulting in high molecular weight which in theory should be regarded as inert structures [2]. According to the Plastics Europe 2018 data report, the world’s plastic production had reached 350 million tonnes in 2017, of which 60 million tonnes were produced by Europe alone (18.5% of world production) [3]. This mass production encompasses a wide variety of materials designed for different applications with plastic packaging being the largest fraction at 39.7%. Plastic packaging in the UK accounts for 2.2 million tonnes of the total production, and this is largely seen in the grocery retail sector (43%) [4,5,6,7].

The large-scale production of plastic packaging has led to many issues such as inappropriate use, incorrect storage and wrong means of transportation causing an estimated (40%) of food waste in developed countries [8]. Improper disposal also has a significant effect on both human health, marine life and the environment. It is estimated that at least 8 million tonnes of plastics leak into the ocean annually [9]. Another common phenomenon is the use of a wide range of chemical additives, low molecular weight fragments, and polymerisation solvents added during plastic production [9] is causing leaching that accumulates over time, creating toxicity issues. Moreover, plastic waste degrades into microplastic particles, or fragments can be ingested by smaller organisms causing considerable stress and damage to wildlife and ecosystems [10].

Materials that come into contact with food during preparation, processing and storage are termed “Food Contact Materials” (FCMs) [11]. These have been shown to behave differently, when put into contact with varying compositions of food, due to various physico-chemical interactions that induce the transfer of their components into food in a process known as migration of substances [12]. These (FCMs) must comply with the existing EU legislation that is enforced by the Food Standard Agency (FSA). The safety of (FCMs) is evaluated by the European Food Safety Authority (EFSA), which safeguards the consumer’s health by controlling the substances used and restricting those with toxic capability [13,14]. The (FCM) regulation (EC) No. 1935/2004 [15] on “materials and articles intended to come into contact with food” sets out requirements for all FCMs [15]. The principles require that materials “do not release their constituents into food at harmful levels, change food compositions, taste or odour in any unacceptable way” [15]. Furthermore, regulation (EC) No. 10/2011 [16] sets out specifications on the use of such substances including migration limits, which specifies the maximum amount of substances allowed to migrate into food during processing or storage [16]. Concerns have been raised regarding the health and safety of these (FCMs) owing to the growing number of studies reporting the migration of substances into food [17].

Poly(styrene) is an aromatic thermoplastic that is easy to mould into different Food Contact Materials, including General Purpose Poly(styrene) (GPPS), High Impact Poly(styrene) (HIPS) and Expanded Poly(styrene) (EPS) [18,19]. (PS) is also amorphous in nature with a glass transition temperature (T_g_) of 90–100 °C due to the stiffening effects of the benzene ring. With a low water absorption, excellent electrical and thermal insulation, plus reasonable chemical resistance, (PS) is considered ideal for food packaging purposes from hinged takeaway containers to Styrofoam cups for soups and hot drinks. The only drawback is that these containers can be effective for a relatively short period of time at mild temperatures (up to 130 °C), or for longer periods of time at refrigerated temperatures (4 °C) [20].

Styrene is an irritant compound of the mucous membranes of the nose and throat, causing wheezing and coughing through repeated long-term inhalation. This can further lead to an onset of depression by affecting the central nervous system with many other symptoms such as headache, sickness, and fatigue. The general population is exposed to styrene in air, drinking water and through consumption of food contained in styrene based (FCMs). This is mainly due to small-sized molecules and the lipophilic nature of styrene, which promotes its absorption and distribution within the body. The benzene ring also serves as a vehicle for penetration through the blood–brain barrier. This occurs mainly through inhalation with ingestion and skin exposure being the other common routes for toxicity [21,22,23]. The World Health Organisation (WHO) has classified styrene as a possible carcinogenic to humans. More than 90% of styrene forms the metabolite styrene 7,8-oxide through a metabolic pathway involving hepatic oxidation by cytochrome P450 [24]. A comprehensive review of this metabolite highlighted a strong correlation to human cancer risk. It stimulates cell replication and leads to cell proliferation [21] as well as inducing single-strand RNA breaks in human white blood cells and promoting cytogenetic damage, which includes chromosomal breaks [24].

As a result of the toxicological profile associated with styrene, extensive studies have been carried out to investigate the level of migration of the monomer into food. Lickly et al. [20] studied the migration from different (PS) foam materials into oil and 8% ethanol simulant at different experimental conditions. It was reported that migration increased as the storage time increased; an increase of 1.9-fold was observed from the 1st to the 4th day of storage, and an increase of 3.1-fold from the 1st to the 10th day [20]. O’Neill et al. [25] carried out tests on milk products in (PS) packaging with 0 to 80% fat content under defined storage conditions. It was found that the migration level of styrene was dependent on the fat content in the milk. It was also concluded that pure water does not simulate the behaviour of milk even at low-fat content, but adding ethanol to the water increases the lipophilic character and thus better mimics the fat-related migration behaviour of milk [25].

The issue of styrene migration has been highlighted over the past 30 years by many published works on the determination of styrene in bottled water and selected foods such as wheat, tomatoes, peaches, olive oil, yogurt and cheese in (PS) packaging by Mason [26], Chiesa et al., [27], Nerin et al., [28,29] and Steele et al. [30].

Nile Red is a lipid soluble fluorescent dye, which has been commonly used in situ for staining of the lipid content of animal cells and microorganisms [31]. Maes et al. (2017) [32] introduced a rapid screening method for microplastics in environmental samples based on adsorption of Nile Red onto plastic surfaces. As a result, microplastic fragments of a range of sizes down to a few µm became clearly visible in blue light, which allowed them to be differentiated from other debris and made it easier to assess micro-plastic abundance [33]. This approach has the potential to highlight microplastics in many other contexts and is being widely adopted for this purpose as awareness of microplastic contamination becomes more widespread.

This work aims to investigate the effects of temperature, food composition and contact times on the migration of the styrene monomer from poly(styrene) food contact materials into food simulants. We present a study of the detection of styrene and poly(styrene) into foodstuffs from some selected food packaging material, varying temperature, and fat content of the foodstuff in question. Increased demand for takeaway and hot food delivery has meant that sources of oligomeric- and micro-plastics from packaging are a potential hazard to the food chain that has thus far been underexplored.

We postulate that temperature and foodstuff composition play a vital role in how much styrene and poly(styrene) are transferred from packaging to food due to an increase in the hydrophobicity of the simulant characteristics from low fat content to high fat content. To that end, techniques such as High-Performance Liquid Chromatography (HPLC), Nile Red staining and microscopy have been used to quantify the amount of oligomeric and polymeric styrene leaches into food under a variety of simulated conditions.

## 2. Materials and Methods

### 2.1. Reagents and Chemicals

Styrene analytical standard (99.9%, contains 4-tert-butylcatechol as stabiliser and polymerisation inhibitor) was purchased from Sigma-Aldrich, Merck Life Science UK Limited, Dorset, UK. Other chemicals: acetic acid glacial (ACS reagent grade assay 99.70%), ethanol 99% denatured with methanol, methanol analytical grade, acetonitrile HPLC grade and Nile Red (N3013 technical grade) were also purchased from Sigma-Aldrich. Polycarbonate Track-Etch Membranes (PTCE, hydrophilic, 25 mm diameter) was purchased from Fisher Scientific Ltd, Loughborough, UK.

### 2.2. Poly(Styrene) Samples

**Figure 1:**
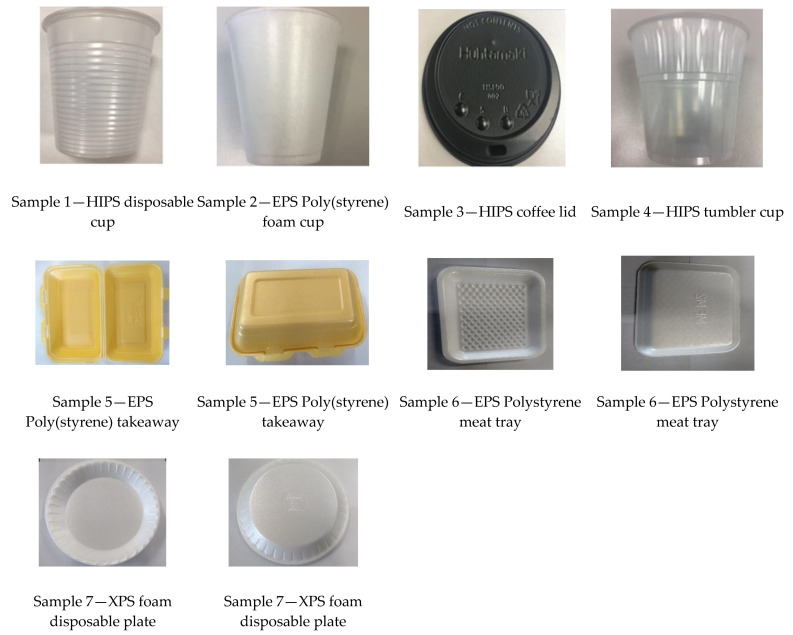
Poly(styrene) samples used for testing.

Images of all poly(styrene) samples are presented in Figure 1. Sample 1: (HIPS) High Impact Poly(styrene) disposable plastic cups. Sample 2: (EPS) Expanded Poly(styrene) foam cup. Sample 3: (HIPS) coffee cup lid. Sample 4: (HIPS) tumbler cup. Sample 5: (EPS) Poly(styrene) takeaway box. Sample 6: (EPS) Poly(styrene) meat tray. Sample 7: (XPS) Extruded Poly(styrene) foam disposable plate. All these materials were purchased online via Delipak UK and Kiel Trade Ltd. venture packaging supplies and distributors.

### 2.3. Food Simulants and Testing Conditions

A food simulant is a chemical with characteristics that imitate food, which is used to model migration of (FCMs) for regulatory testing purposes. The food simulants chosen for this study are based on recent regulations provided by the EU Commission (European Union, EU) No. 10/2011 [16] and presented in Table 1. Moreover, the testing conditions were chosen based on the recommended standardised testing conditions outlined in the EU-Directive 10/2011, whereby the materials for testing shall be placed in contact with the food simulant in a manner representing the worst foreseeable conditions of use as regards contact time and contact temperature. Samples 1–4 chosen for testing are those commonly used in high temperature applications at 100 °C [1] and therefore, considering the intended use, these samples were placed in a pre-heated water bath to 60 °C. This is due to the azeotropic temperature of ethanol being less than 100 °C. The food container Samples 5–7 were more varied in their use and therefore the following testing conditions were employed: 2 h at 60 °C, 2 h at 70 °C and 240 h at 5 °C. Each sample was covered with two layers of cling film and a watch glass to prevent evaporation of simulants.

### 2.4. HPLC Conditions

Quantitative HPLC analysis was conducted using an Agilent 1260 Infinity II HPLC with a wavelength of 245 nm. Injection volume was up to 50 µL with a flow rate of 1 mL/minute, and a run time of 3 min. Column used was Phenomenex C18, size 250 × 3.00 mm (5 micron), type: AQUA 5u C18 125A, P/NO: 00G-4299-Y0. Mobile phase used was acetonitrile and water (75:25 *v*/*v*), under isocratic conditions [33].

### 2.5. Experimental Procedure

#### 2.5.1. Preparing the Calibration Curves

**Figure 2:**
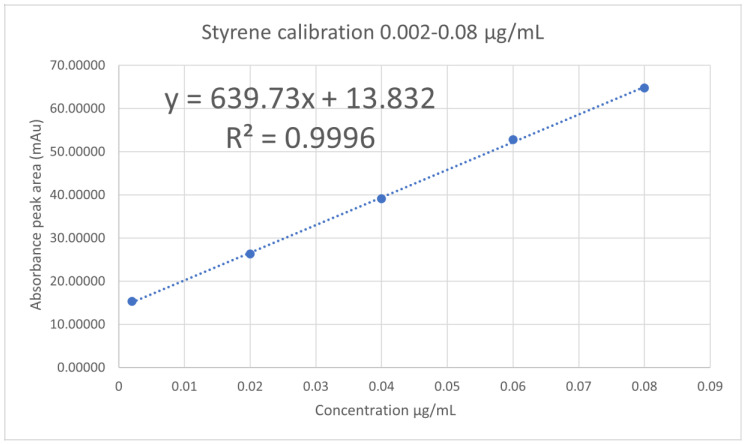
An example of calibration curve of styrene 0.002–0.08 µg/mL.

Styrene stock solution was prepared using the styrene analytical standard ampoule (1.1 mL) into a 10 mL volumetric flask and made up with methanol to give a concentration of 100 µg/mL. This was further diluted into working solutions of 10 µg/mL and 1 µg/mL which were both used to make up a wide range of standard solutions (at least set of 13 standards) of different concentrations 0.002 µg/mL–10 µg/mL. Several calibration curves over different days were produced and selected calibration curves are presented in Figure 2 (and Figures S3 and S4 in the SI), with selected summaries of the validation data for calibration curves shown in Table 2.

#### 2.5.2. Sample Preparation

Different Poly(styrene) samples were cut into 2–3 × 2–3 cm pieces weighing around 2.0 g each piece and placed into a 200 mL beaker. The simulants were prepared according to their concentrations as presented in Table 1 and made up to a 100 mL with distilled water. The (PS) samples were then immersed in the 100 mL of the simulant and covered with 2 layers of cling film and a watch glass and tested under a set of different conditions outlined in Section 2.3.

#### 2.5.3. Nile Red

The Poly(styrene) samples were removed, and the remaining solution filtered under vacuum onto polycarbonate track-etch membranes, which were then placed onto a petri dish, covered, and left to dry in a warm area for 24 h. Nile Red was made up to a working solution of 1µg/mL in methanol and 2–3 drops were added onto each filter, transferred onto a microscopic slide, covered with a clean cover slip and left in the darkness for 10 min. All samples were then viewed under an Axio Observer Z1/7 microscope with an EC Plan Neofluar 10 × 0.30 m27 objective lens at an emission of 636 nm and an excitation of 559 nm; and imaged with an LSM800 MA Pmt2 imaging device [32].

## 3. Results

### 3.1. Drinking Cup Samples 1–4

The regression equations from the several calibration curves were used to calculate the levels of styrene within the food simulants. The results of styrene migration from the various samples are presented in Table 3 and Table 4.

**Figure 3:**
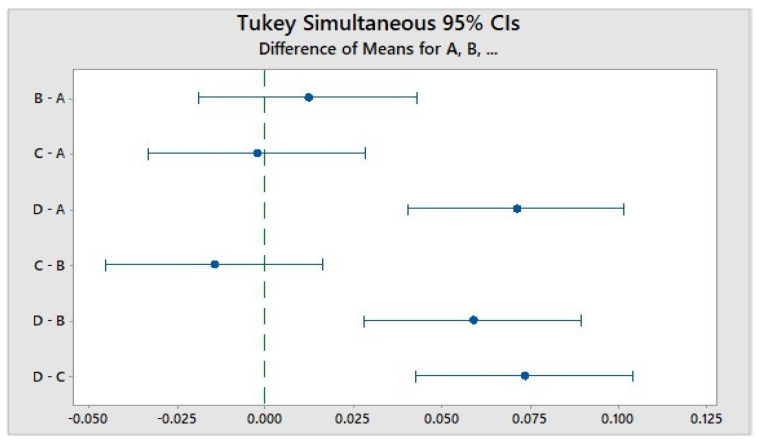
Tukey Diagram representing ANOVA comparison of simulants A, B, C and D.

Initially the samples were to be heated up to 100 °C; however, shrinking and melting of the Poly(styrene) samples were observed and therefore the temperature was reduced to about 70 °C, whereby the samples were not visibly affected. ANOVA statistical analysis was used to compare the difference between the (PS) samples and simulants. There was no statistically significant difference between any of the cups (*p* = 0.920) at the 99% confidence interval (*p* < 0.01). However, when the mean values for each simulant were compared there was a statistically significant difference at the 95% confidence interval between simulant D and simulants A/B/C. This can be seen in the Tukey diagram in Figure 3, whereby lines that do not cross zero demonstrate a statistically significant difference. Interpretation of this data supports the findings that higher fat content induces more styrene leaching as ethanol 50% represents dairy foods.

### 3.2. Food Container Samples 5–7

**Figure 4:**
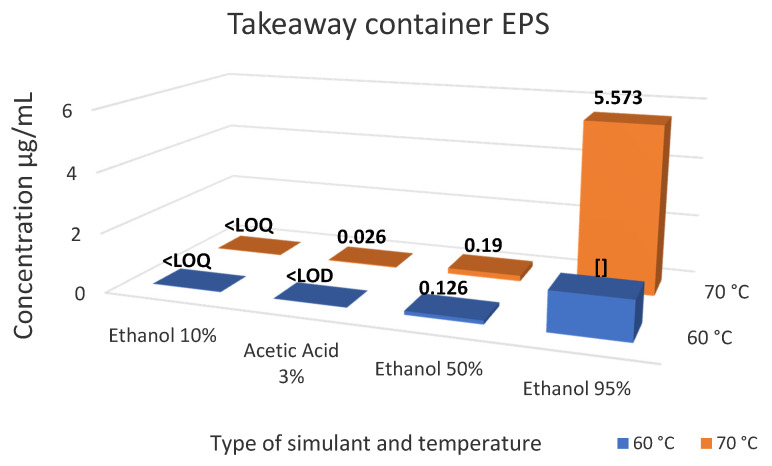
Summary of styrene migration results for Sample 5—Takeaway Container.

**Figure 5:**
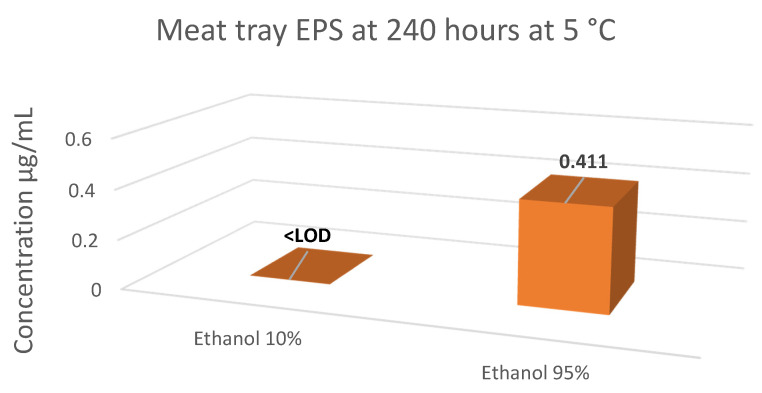
Summary of styrene migration results for Sample 6—Meat Tray.

**Figure 6:**
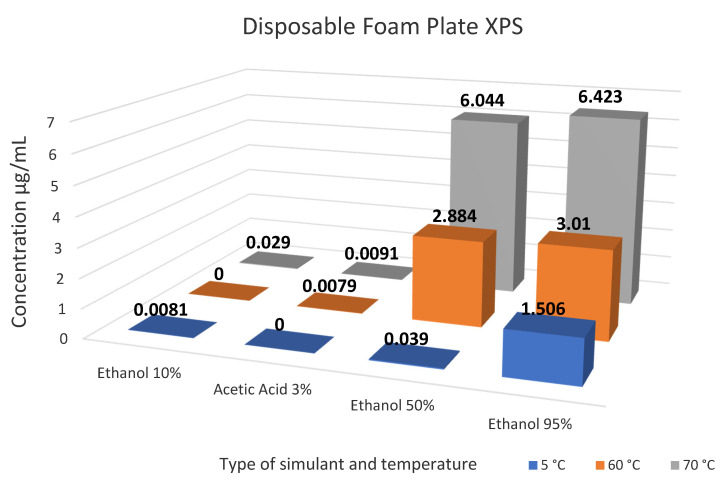
Summary of styrene migration results for Sample 7-Disposable Foam Plate.

The regression equations from the calibration curves were used to calculate the levels of styrene within the food simulants. Results for the migration of styrene for Samples 1–4 and Samples 5–7 are summarised in Table 3 and Table 4, respectively with Figure 4, Figure 5 and Figure 6 represent the level of styrene migration in samples 5–7. ANOVA statistical analysis was used to compare the means of three or more groups of data, and t-test analysis was used when comparing the means of two groups. This was carried out on each individual sample to determine the significance of changing the temperature, simulant or both on the level of migration observed in the results. All significant values were tested at 95% confidence level *p <* 0.05.

Sample 5, the takeaway containers, were originally tested for 2 h at 70 °C and 100 °C; however, due to ethanol’s azeotropic boiling temperature being lower than 100 °C, those simulants were instead tested at 60 °C and 70 °C as shown in Table 4. The lowest levels of migration were found in ethanol 10% and acetic acid 3%, where they were below the values of LOD and/or LOQ. The highest migration values were found in ethanol 95%, whereby samples at 70 °C showed a higher migration relative to the samples tested at 60 °C (5.57 vs. 1.31 µg/mL). ANOVA single factor analysis comparing the significance of changing the simulant and increasing the temperature was conducted to determine whether there is a statistically significant relationship between the variables. An increase in fat characteristics of the simulant showed a statistically significant increase in migration F(3, 28) = 17.7, *p* = 1.19 × 10^−6^, which was particularly evident when comparing the change between the migration levels of ethanol 10% acetic acid 3% with ethanol 50% and 95%. However, the increase in temperature from 60 °C to 70 °C (a temperature closer to its boiling point) did not show statistically significant differences—t(17) = −1.62, *p* = 0.124.

Sample 6, the meat trays, were kept at 5 °C for 240 h (10 days), as meat trays are almost never used in heated conditions. Other temperatures were not tested; however, two contrasting simulants were used instead. For the lower fat content, ethanol 10%, migration was lower than the detection limit. The higher fat content simulant ethanol 95% showed a higher migration level of 0.411 µg/mL; however, this was still a relatively low migration level due to the temperature conditions.

Sample 7, the disposable plates were tested under all three different conditions mentioned in Samples 5 and 6. The level of migration follows a similar pattern, whereby the concentration of styrene increases as the fat content in the simulant increases F(3, 36) = 9.98, *p* = 6.27 × 10^−5^ and as the temperature increases F(2, 37) = 11.52, *p* = 0.0001 

### 3.3. Calibration Curves

Linearity was observed using 5 concentrations in all ranges. The correlation coefficient *R*^2^ value was 0.9996–0.9998. Selected calibration curves of styrene standards 0.002–0.08 µg/mL, 0–2 µg/mL and 2–10 µg/mL as previously mentioned are presented in Figure 2 (and Figures S3 and S4 in SI).

**Figure 7:**
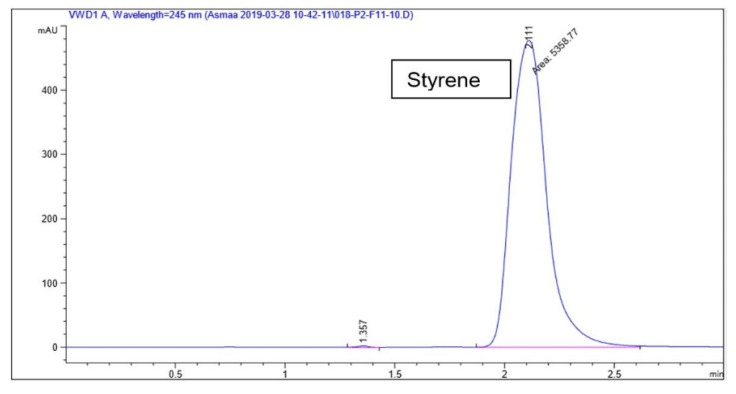
HPLC chromatograph of methanol solution spiked with 10 µg/mL styrene, RT 2.1–2.3.

Data is presented from the HPLC chromatographs of styrene analytical solution used in making up concentrations for the calibration curves. Specificity was evaluated by comparing the spiked styrene solution with blank runs of methanol. A peak representing 10 µg/mL of styrene is seen at the retention time 2.1–2.3 shown in Figure 7. HPLC method was validated through the International Council for Harmonisation of Technical Requirements for Pharmaceuticals for Human Use (ICH) [34]. Linearity, range, specificity, accuracy, precision, Limit of Detection and Quantification (LOD, LOQ) are all taken into account.

Calibration curves: A regression analysis was performed examining the linearity and fit of the two variables generated a regression equation of *y* = 633.113*x* ± 2.529 and a correlation coefficient (*r*^2^) of 0.9996 showed a linear fit. A regression analysis of variance evaluated the significance of this linear relationship of F(1,70) = 21,903.27, *p <* 0.05 indicating a sensible linear model. Accuracy calculated through percentage recovery of samples and its Relative Standard Deviation (RSD) was found to be 98.3 ± 3.29%.

A regression analysis examining the linearity and fit of the two variables generated a regression equation of *y* = 524.81*x* ± 80.51 and a correlation coefficient (*r*^2^) of 0.9996 showing a linear fit. A regression analysis of variance evaluated the significance of this linear relationship of F(1,43) = 107693.31, *p <* 0.05 indicating a sensible linear model. Accuracy calculated through percentage recovery of samples and its Relative Standard Deviation (RSD) was found to be 100.34 ± 1.95%. Precision evaluated by RSD for repeatability (intra-day) and intermediate precision (inter-day) gave RSD values of less than 1% indicating good precision.

### 3.4. Nile Red

**Figure 8:**
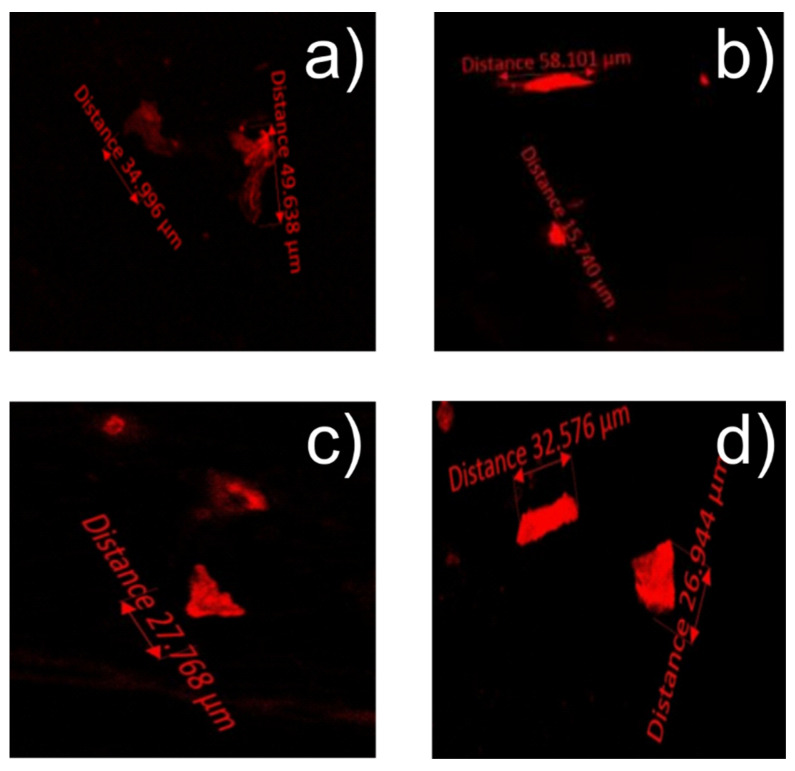
Representative Nile Red staining images showing microplastic ingress from the EPS poly(styrene) meat tray at 5 °C. (**a**) 10% Ethanol, (**b**) 95% Ethanol, (**c**) 50% Ethanol and (**d**) 3% acetic acid.

Three types of Poly(styrene) Samples 5–7 were used for the Nile Red staining, cut and stained following procedures in Section 2.5.2 and Section 2.5.3. Squares of 2–3 × 2–3 cm were prepared by cutting (PS) into clean edged pieces using a scalpel and ensuring no loose ends being generated (see Figure S1). A control experiment with square plastic samples was also performed using the methods above to demonstrate no plastic fragment was generated from the cutting process during experimental preparation (see Figure S1). Each sample was then viewed under an Axio Observer Z1/7 microscope with an EC Plan Neofluar 10 × 0.30 m27 objective lens at an emission of 636 nm and an excitation of 559 nm, using an LSM800 MA Pmt2 imaging device to record images shown in Figure 8 and MP size ranges in Table 5. It seems that both 70 °C temperature and 50%–95% ethanol produced the most microplastics. Additional Nile Red images are also presented in Tables S1–S3 with different simulants.

## 4. Discussion

This work aimed to investigate the level of migration of styrene in 7 Poly(styrene) food and drink samples: HIPS, EPS and XPS types. Water as a simulant was replaced by Ethanol 10% when testing food EPS and XPS Samples 5–7, because fat-free foods do not entirely behave like water.

In Samples 1–4, the highest levels of styrene was detected in the range (0.0406–0.111 µg/mL) and found to be present in simulant D, which represent fatty foods. This was supported by the statistical analysis carried out, which showed there was a significant difference in migration between simulant D and the other stimulants. All poly(styrene) samples generally leached less in water (0.00740–0.00390 µg/mL) and ethanol 10% (0.000400–0.00100 µg/mL), as would be expected.

Similarly, in the EPS food container samples, many of the undetected values were those of samples in 10% ethanol stimulant and even more so when the temperature was set at 5 °C. The lowest detectable level of styrene was in the range of (0.0121–0.0940 µg/mL) in ethanol 10% and/or acetic acid 3%. When compared with the samples tested in 95% ethanol simulant that represent foods with lipophilic properties of both dairy and non-dairy fatty foods, the range was within (0.190–6.42 µg/mL). The styrene level detected increased as the fat content increased, represented by an increase of form 10% to 95% ethanol simulants, which was seen to be statistically significant in all samples. This was also seen as the temperature increased in all the samples. Both factors had a combined effect on the migration of styrene, reflecting the trend observed in previous studies [20,26,35].

Water is polar and the hydrophobic nature of the monomer could have resulted in insolubility in water and ethanol 10%. Sample 2 (EPS Styrofoam cup) generally had the lowest concentration of monomer detected for all the simulants tested. EPS is comprised of approximately 95% gaseous blowing agent and 5% Poly(styrene) [35]. The low level detected could be attributed to the relatively small percentage of (PS) contained within the structure. However, an alternative factor to consider is the high impact resistance and strong thermal insulation properties associated with EPS. Impact resistance is the resistance of a material to fracture under sudden impact, where a high resistance results in low energy absorption [36]. Thermal insulation is the reduction of heat transfer between objects that are heated and it provides a region of insulation in which thermal conduction or radiation is reflected rather than absorbed. Both of these physical features may have resulted in the reduced transfer of heat energy across the polymers leading to less styrene migration.

Furthermore, polymerisation impurities can be present on the plastic surface. These may be low molecular weight polymer fragments that further increased the levels of migration [37]. Another important factor is that the migration of styrene has been observed to vary according to the interaction with the simulant and change in temperature. When a thermoplastic polymer is heated, the energy of the polymer chains increases allowing the chains to slide past each other and overcome inter-molecular forces and causing a degree of melting [38]. The likelihood of migration increasing when the temperature changed from 5 °C to 70 °C could have led to a greater pliability due to further weakening of Poly(styrene) chain interactions [39].

It has also been recognised that the food simulants used may have had an effect on the transfer of styrene because it is a non-polar molecule and therefore is more likely to transfer to similar non-polar simulants such as the ethanol 95% sample. The type of (PS) used in this work may also have contributed to the level of migration. An example is that EPS is a highly amorphous and porous material; therefore, it is likely that the sorption of high ethanol solutions into the matrix of the polymer resulted in swelling of styrene into the simulant [16].

All levels detected in this study have been well below the migration limit set out by the EU commission regulation 10/2011 on plastic materials and articles intended to come into contact with food, outlining that a migration limit for unspecified articles such as styrene should be less than 60 mg/kg [13]. Article 17 of EU 10/2011 regulation states that, in containers, containing less than 500 millilitres or grams or more than 10 litres, a value of migration shall be expressed in mg/kg applying a surface to volume ratio of 6 dm^2^/kg of food.

However, this may not be the only dietary intake from (PS) food packaging, as microplastic detected by Nile Red staining indicated that a small amount of microplastics is ingested leading to prolonged exposure times to plastic, albeit an incredibly small amount compared to the bulk container. The jagged nature of the fragments imaged suggests that they have broken off the containers, rather than, for instance, being aggregated “blobs” of diffusing oligomers (which would have low T_g_ and would be soft). This needs more detailed investigation, however. The effects of ingestion of microplastics are currently very poorly understood, even in small model organisms, but cumulative levels from many sources, including food, drinking water (mains or bottled), etc., could be quite high. In their review, Rainieri and Barranco [40] highlighted the risk not only of the migration and presence of microplastics in food, but also in food additives with chemical contamination absorbed by the microplastics affecting both animals and environmental health. The study also urges more work to be carried out in order to evaluate risk assessment of microplastics in foodstuff and their effect on human health and this is certainly an area that should be studied in much more detail.

In the U.S., the Federal Food and Drug Administration (FDA) has stated that an Acceptable Daily Intake (ADI) value of styrene is calculated to be 90,000 µg/person/day [41]. However, exposures to styrene extend beyond food ingestion and other routes include levels in air that could lead to a prolonged accumulation of styrene in the body through inhalation over time. Another factor could be cigarette smoking, a major source of styrene exposure that may also accumulate in the body over time. Hence, although the levels of styrene migration are below the level of Overall Migration Limits (OML) set by the EU [40], real life exposures may very well differ in their quantity and intake.

## 5. Conclusions

Poly(styrene) cups showed a relatively low level of styrene migration with the highest being 0.110 µg/mL, whereas food containers showed a much higher level of styrene leaching of up to 6 µg/mL. This could be due to an increase in the hydrophobicity of the simulants’ characteristics from low to high fat content and the increase in the testing temperatures from 5 °C to 70 °C. Statistically, results showed that the migration level of styrene has increased, the oil content being 95% ethanol. This means increasing the amounts of fat content in food, combined with increasing temperatures and longer exposure to the packaging, significantly increased the level of migration and thus ingestion from food.

ANOVA statistical analysis is used to compare the means of three or more groups of data, whereas t-test analysis is used to compare the means of two groups. This was carried out on each individual sample to determine the significance of changing the temperature, simulant type, or both, on the level of migration observed in the results. All significant values were tested at 95% confidence level *p <* 0.05, concluding that fat content and high temperatures were found to significantly increase the level of styrene migration.

HPLC was used to detect styrene migration in the range of (0.0004 µg/mL to 6.423 µg/mL) across all food and drink packaging samples tested. Although the migration levels in this investigation did not exceed the safety limits of (10 mg/dm^2^) set out in regulatory documents by the European Union 10/2011 legislation, the cumulative exposure to styrene from food packaging and other routes should not be overlooked.

From the HPLC chromatograph of methanol solution spiked with 10 µg/mL styrene, RT seems to be consistent at around 2.1–2.3 for styrene. (See Figures S5 and S6 in SI). Other peaks could be assigned to 4-*tert*-butylcatechol, a styrene stabilizer used to inhibit polymerization into (PS). Contamination, polymerization, or change of column can also change the elution time and add extra peaks. Moreover, styrene can behave differently in different solvents which may lead to different retention times (RT). Another difficulty faced with HPLC analysis is that distorted peaks can also be a result of a mix up with background noise. Moreover, introducing new columns or stationary phases species can interact with the analyte in an unintentional manner in what is called ‘silanol’ species forming.

The ability of Nile red to detect leaching Poly(styrene) microplastic particles was also seen to be a successful method. Nile Red stain was reported [26] to be adsorbed to polymeric materials and fluorescences under specific wavelengths of incident light. These particles were measured at between 6 µm and 104 µm in size, with the highest migration (2–10 per cm^2^) observed from a Poly(styrene) in simulant 50–95% ethanol at 70 °C. 

This did not include any consideration of additional microplastic migration due to behaviour, such as use of cutlery, so in practice levels could be much higher. The desirability of phasing out Poly(styrene)-based packaging is well understood, but in reality, it is likely to be in use for many more years, hence more detailed exposure estimates are still of great relevance and microplastic shedding is an emerging issue that should be evaluated much more extensively, particularly as alternatives to Poly(styrene) are sought and introduced.

## Figures and Tables

**Table 1:** Food simulants used for migration testing.

Simulants	Contact Foods
A-10% aq. Ethanol/distilled Water	Aqueous foods (pH *>* 4.5)
B-3% aq. Acetic acid	Acidic food (pH *<* 4.5)
C-50% aq. Ethanol	Diary food products
D-95% aq. Ethanol	High fat content foods

**Table 2:** Selected summaries of validation data for calibration curves.

Parameters	Value
Accuracy	98.3 *±* 3.29
Slope	633.113
Intercept	12.529
Linearity range	0.02–0.08 µg/mL
Correlation coefficient (r)	0.9996
Standard Error	1.6650
LOD	0.0086 µg/mL
LOQ	0.0263 µg/mL
**Parameters**	**Value**
Accuracy	100.34 *±* 1.95
Slope	524.81
Intercept	80.51
Linearity range	2–10 µg/mL
Correlation coefficient (r)	0.9996
Standard Error	10.608
LOD	0.066 µg/mL
LOQ	0.202 µg/mL

**Table 3:** Summary of styrene migration from Sample 1–4.

Article	Food Simulant	Time (h)	Temperature	Concentration µg/mL
Sample 1: HIPS Poly(styrene) cup	A-Water	1 h	60 °C	0.0074
	B-Acetic acid 3%	1 h	60 °C	0.0121
	C-Ethanol 10%	1 h	60 °C	0.0004
	D-Ethanol 50%	1 h	60 °C	0.0773
Sample 2: EPS Styrofoam cup	A-Water	1 h	60 °C	0.0074
	B-Acetic acid 3%	1 h	60 °C	0.0025
	C-Ethanol 10%	1 h	60 °C	0.0080
	D-Ethanol 50%	1 h	60 °C	0.0779
Sample 3: HIPS Coffee lid	A-Water	1 h	60 °C	0.0039
	B-Acetic acid 3%	1 h	60 °C	0.0172
	C-Ethanol 10%	1 h	60 °C	0.0010
	D-Ethanol 50%	1 h	60 °C	0.1105
Sample 4: HIPS Tumbler cup	A-Water	1 h	60 °C	0.0039
	B-Acetic acid 3%	1 h	60 °C	0.0167
	C-Ethanol 10%	1 h	60 °C	0.0033
	D-Ethanol 50%	1 h	60 °C	0.0406

**Table 4:** Summary of migration results for Samples 5–7.

Article	Food Simulant	Time (h)	Temperature	Concentration µg/mL
Sample 5: EPS	Ethanol 10%	2	60 °C	0.00880 = <LOQ
Takeaway Container		2	70 °C	0.00950 = <LOQ
	Acetic Acid 3%	2	60 °C	0.00480 = <LOD
		2	70 °C	0.0260
	Ethanol 50%	2	60 °C	0.126
		2	70 °C	0.190
	Ethanol 95%	2	60 °C	1.31
		2	70 °C	5.57
Sample: 6 EPS	Ethanol 10%	240	5 °C	0.000300 = <LOD
Meat Tray	Ethanol 95%	240	5 °C	0.411
Sample 7 XPS	Ethanol 10%	240	5 °C	0.00810
Disposable Foam		2	70 °C	0.029
Plate	Acetic Acid 3%	2	60 °C	0.0079
		2	70 °C	0.0091
	Ethanol 50%	240	5 °C	0.0390
		2	60 °C	2.88
		2	70 °C	6.04
	Ethanol 95%	240	5 °C	1.51
		2	60 °C	3.01
		2	70 °C	6.42

**Table 5:** Microplastic particles size and range per cm^2^ measured using Nile Red staining indicative of plastic leaching in Samples 5–7.

Microplastic Size in μm	Poly(Styrene) Type	Simulant and Temperature	Microplastic Pieces Per cm^2^
52.9	EPS takeaway container	3% Acetic acid at 60 °C	1–6
17.6	EPS meat tray	50% Ethanol at 60 °C	2–6
6.40	EPS meat tray	50% Ethanol at 70 °C	4–10
104	EPS meat tray	95% Ethanol at 70 °C	2–10
58.9	XPS disposable plate	50% Ethanol at 5 °C	1–4
11.8	XPS disposable plate	95% Ethanol at 5 °C	1–2

## Supplementary Materials

The following are available online at https://www.mdpi.com/article/10.3390/foods10051136/s1, Table S1. Nile red staining indicative of plastic leaching in Samples 5–7 tested after 10 days at 5 °C, Table S2. Nile red staining indicative of plastic leaching in Samples 5–7 tested after 2 hours at 60 °C, Table S3. Nile red staining indicative of plastic leaching in Samples 5–7 tested after 10 days at 70 °C, Figure S1. Control experiment, Figure S2. Images of clear of PCTE membrane from the microplastics - control experiment using Axio Observer Z1/7 microscope with an EC Plan Neofluar 10 × 0.30 m27 objective lens at an emission of 636 nm and an excitation of 559 nm; using an LSM800 MA Pmt2 imaging device, Figure S3. Calibration curve for styrene 0–2 µg/ml, Figure S4. Calibration curve for styrene 2–10 µg/mL, Figure S5 Chromatograph of a standard styrene solution with concentration 0.8 µg/mL, Figure S6. Chromatograph of a standard styrene solution with concentration 10 µg/mL, Figure S7: HPLC Migration of styrene in 3% acetic acid for 2 hours at 70 °C, Figure S8: HPLC Migration of styrene in 10% ethanol for 240 h at 5 °C, Figure S9: HPLC Migration of styrene in 10% ethanol for 2 hours at 70 °C, Figure S10: HPLC Migration of styrene in 50% ethanol for 2hours at 70 °C, Figure S11: HPLC Migration of styrene in 95% ethanol for 2 hours at 70 °C.
