# Supplementary material for: An Insight into the Growing Concerns of Styrene Monomer and Poly(Styrene) Fragment Migration into Food and Drink Simulants from Poly(Styrene) Packaging
Source: Foods. 2021 May 20;10(5):1136. doi: 10.3390/foods10051136 (PMC8160766; doi:10.3390/foods10051136)
Supplement: Supplementary file 1 [file foods-10-01136-s001.zip › foods-1173510-supplementary.pdf]

Table of Contents 2

Nile red images 2
Table S1 2
A: EPS polystyrene meat tray at 5°C 2
B: EPS polystyrene take away at 5°C 3
C: XPS polystyrene foam disposable plate at 5°C 4
Table S2. 5
A: EPS polystyrene meat tray at 60°C 5
B: EPS polystyrene take away at 60°C 6
C: XPS polystyrene disposable plate at 60°C 7
Table S3. 8
A: EPS polystyrene meat tray at 70°C 8
B: EPS polystyrene take away at 70°C 9
C: XPS polystyrene disposable plate at 70°C 10
Figure S2. 12
Figure S3. 12
Figure S4 12
Figure S5a 13
Figure S5b 13
Figure S6a 14
Figure S6b 14
Figure S6c 15
Figure S6d 15
Figure S6e 16

Nile Red Images

Table S1. Nile red staining indicative of plastic leaching in samples 5–7 tested after 10 days at 5 °C

A: EPS polystyrene meat tray at 5 °C.

10% Ethanol	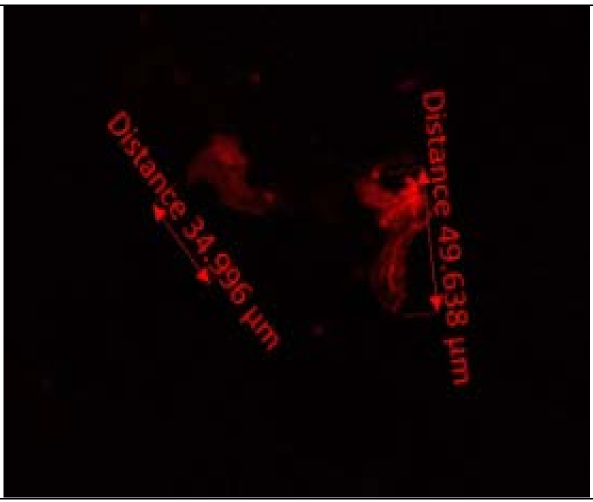	95% Ethanol	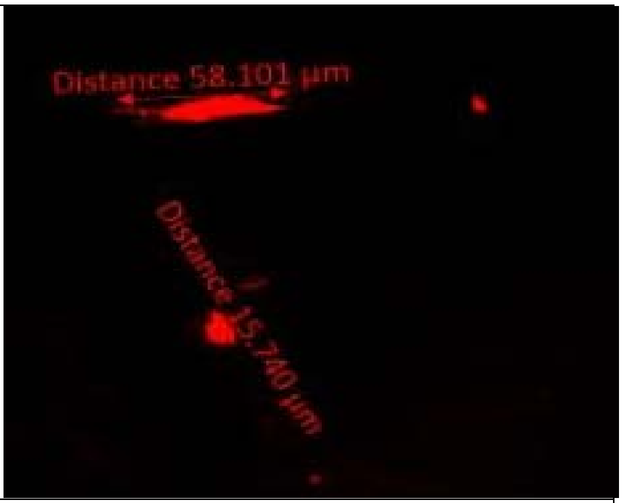
50% Ethanol	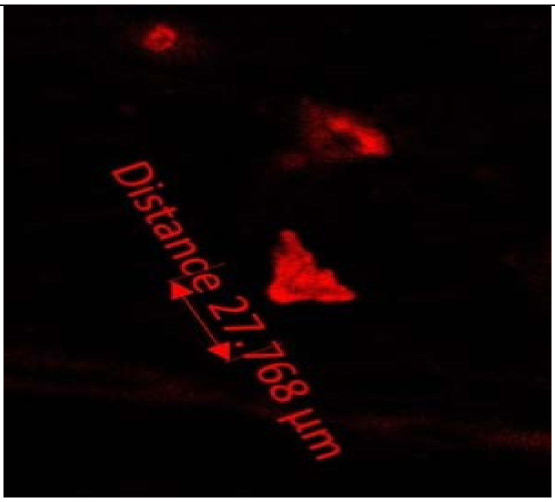	3% Acetic Acid	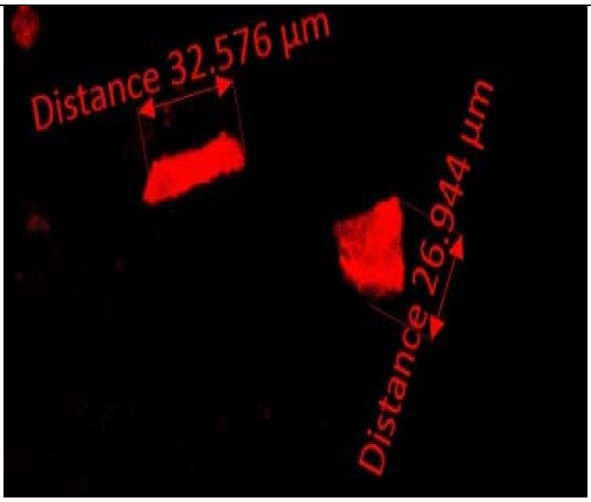

B: EPS polystyrene take away at 5 °C.

10% Ethanol	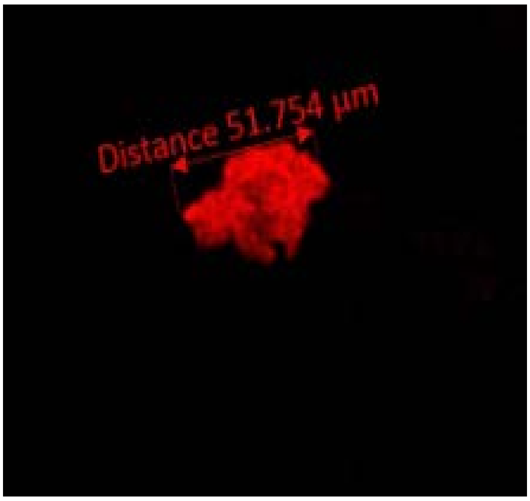	95% Ethanol	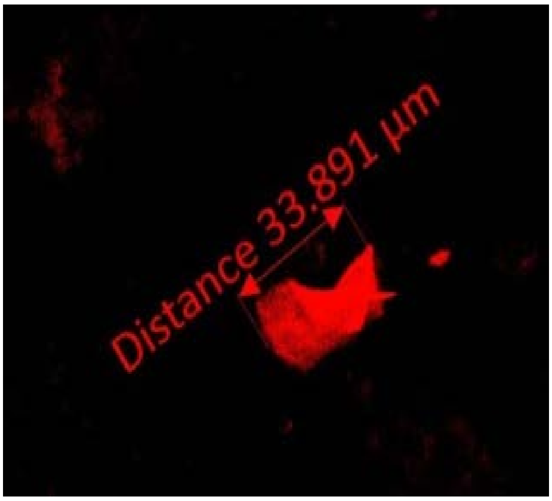
50% Ethanol	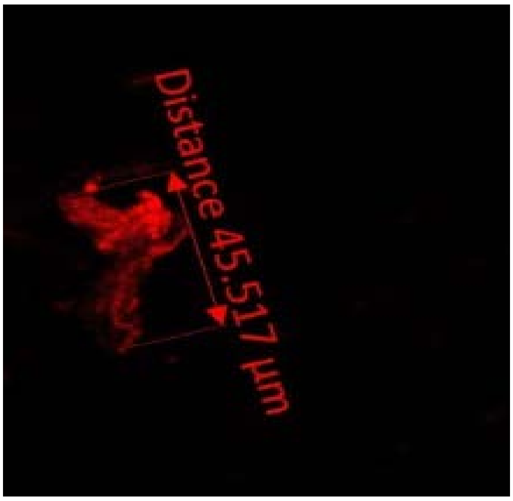	3% Acetic Acid	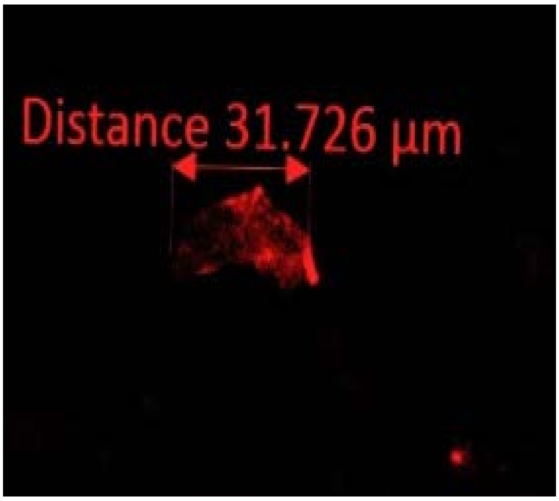

C: XPS polystyrene foam disposable plate at 5 °C

<table>
<tr><td>10% Ethanol</td><td>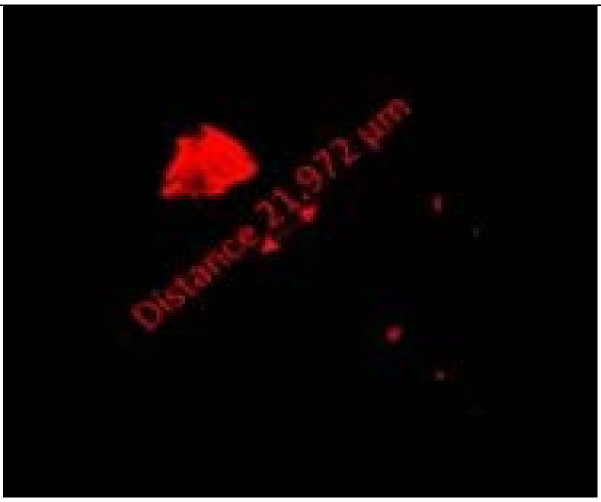</td><td>95% Ethanol</td><td>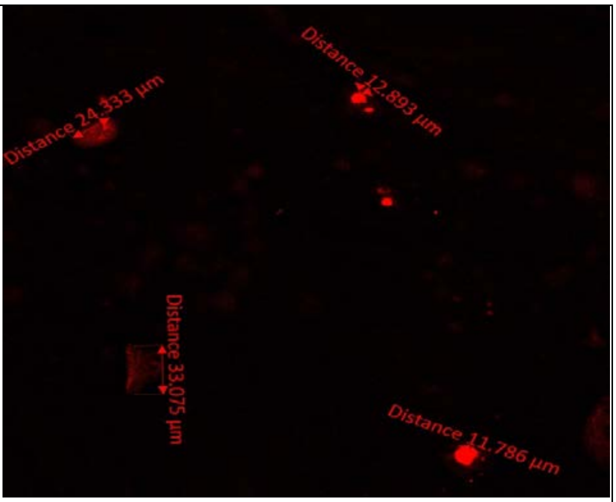</td></tr>
<tr><td></td><td></td><td></td><td></td></tr>
<tr><td>50% Ethanol</td><td>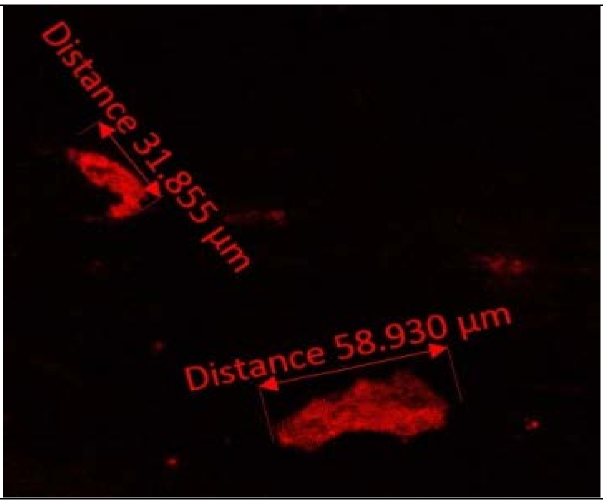</td><td>3% Acetic Acid</td><td>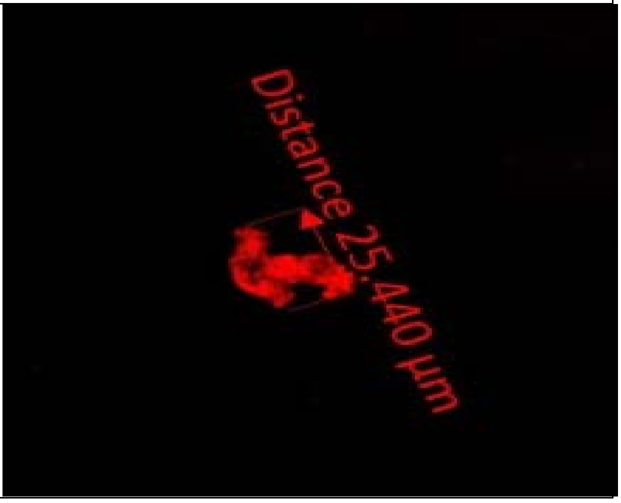</td></tr>
</table>

Table S2. Nile red staining indicative of plastic leaching in samples 5–7 tested after 2 hours at 60 °C

A: EPS polystyrene meat tray at 60 °C.

<table>
<tr><td>10% Ethanol</td><td>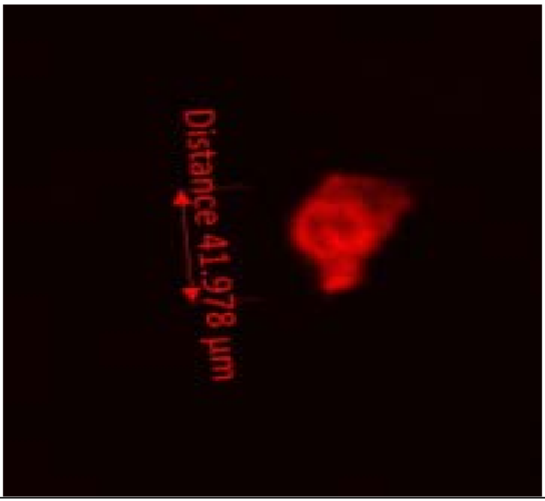
</td><td>95% Ethanol</td><td>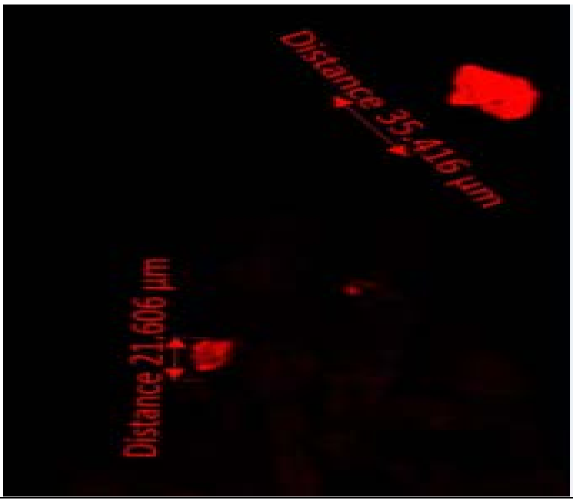
</td></tr>
<tr><td></td><td></td><td></td><td></td></tr>
<tr><td>50% Ethanol</td><td>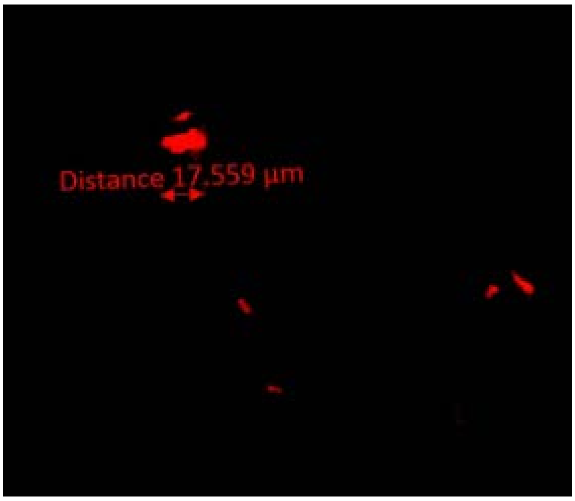
</td><td>3% Acetic Acid</td><td>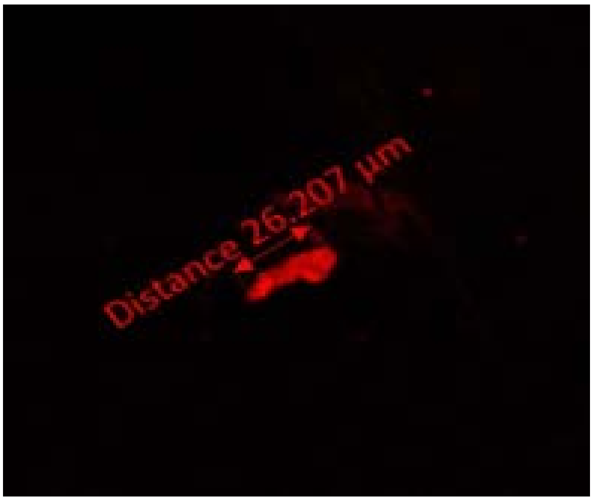
</td></tr>
</table>

B: EPS polystyrene take away at 60°C

<table>
<tr><td>10% Ethanol</td><td>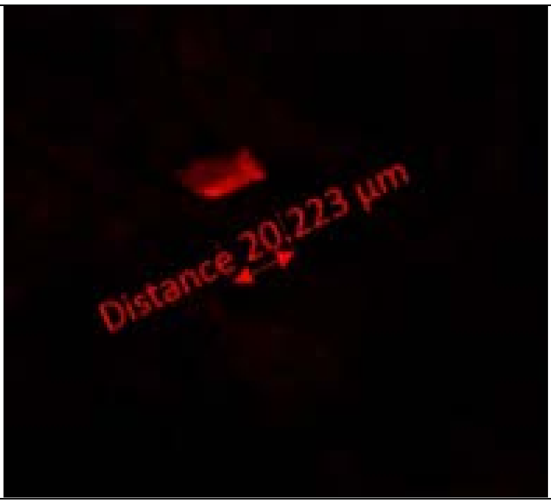
</td><td>95% Ethanol</td><td>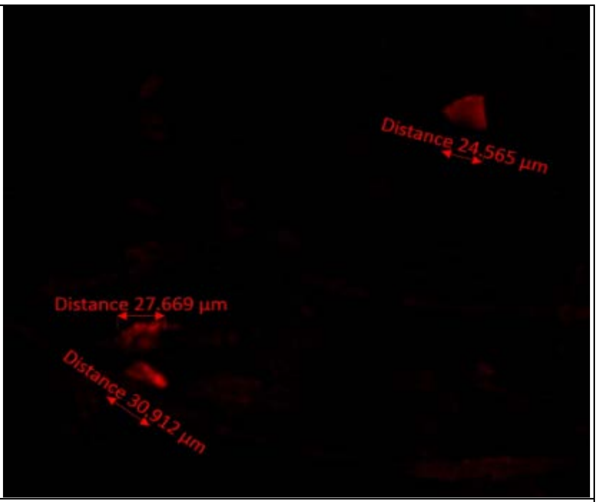
</td></tr>
<tr><td></td><td></td><td></td><td></td></tr>
<tr><td>50% Ethanol</td><td>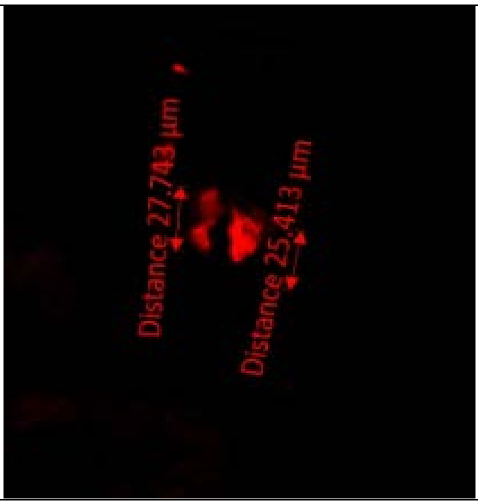
</td><td>3% Acetic Acid</td><td>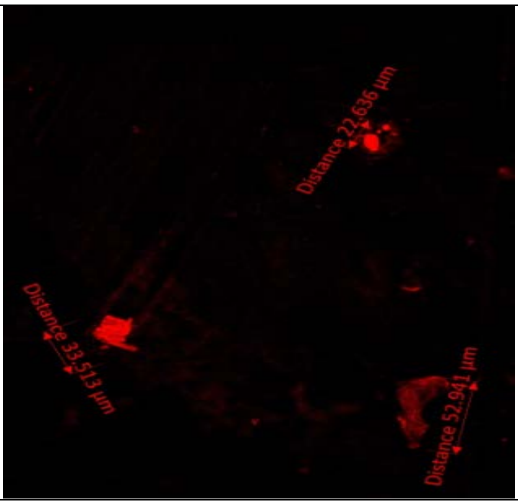
</td></tr>
</table>

C: XPS polystyrene disposable plate at 60 °C.

10% Ethanol	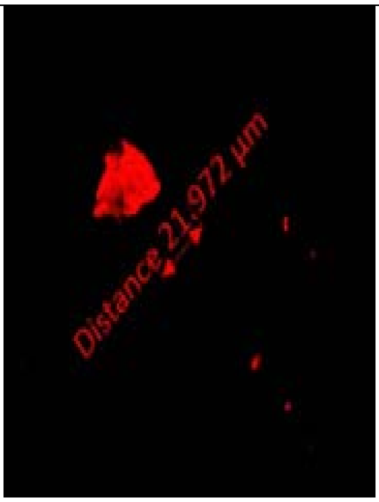	95% Ethanol	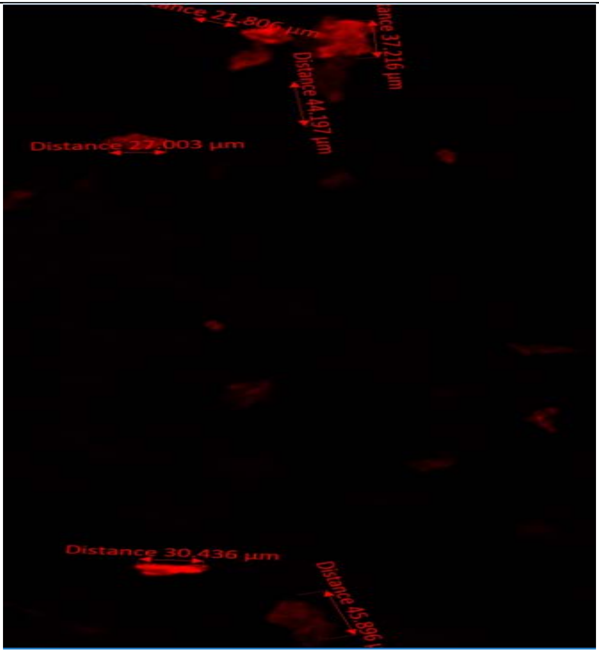
50% Ethanol	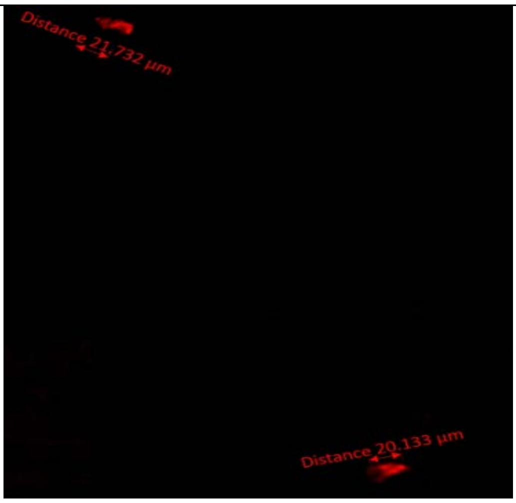	3% Acetic Acid	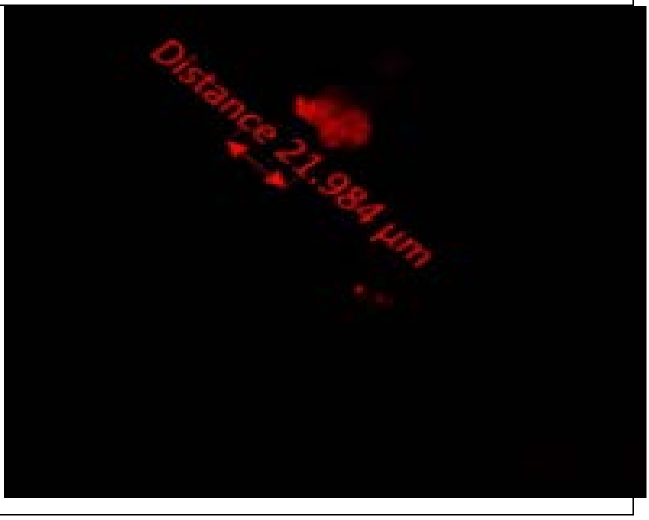

Table S3. Nile red staining indicative of plastic leaching in samples 5–7 tested after 10 days at 70 °C.

A: EPS polystyrene meat tray at 70 °C.

10% Ethanol	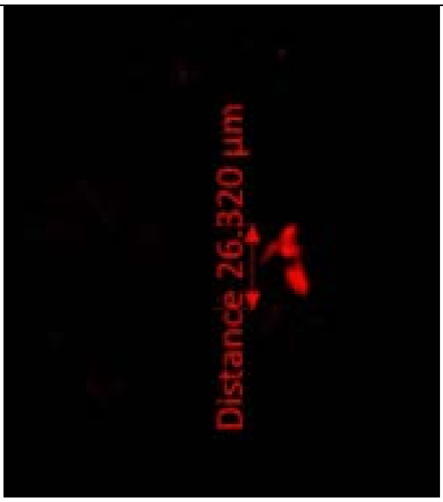	95% Ethanol	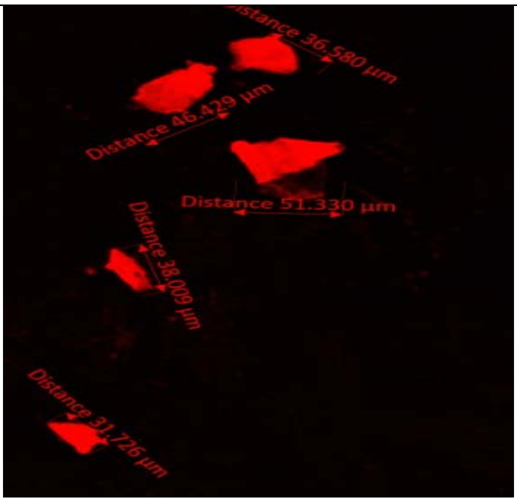
50% Ethanol	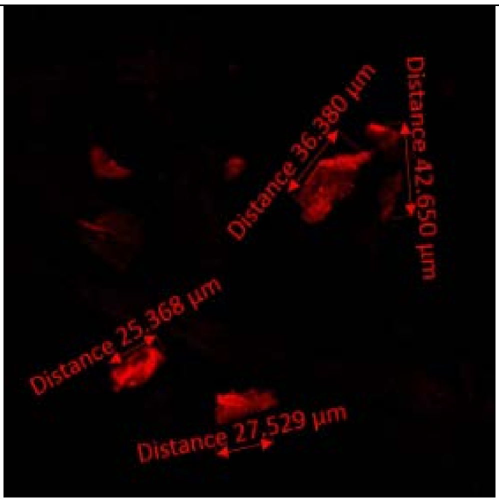	3% Acetic Acid	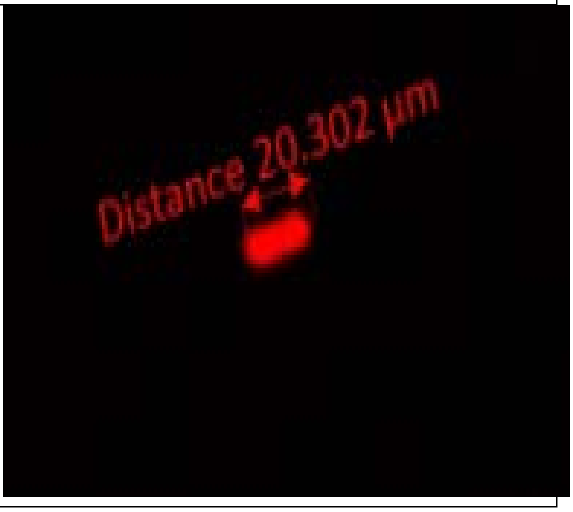

B: EPS polystyrene take away at 70 °C.

10% Ethanol	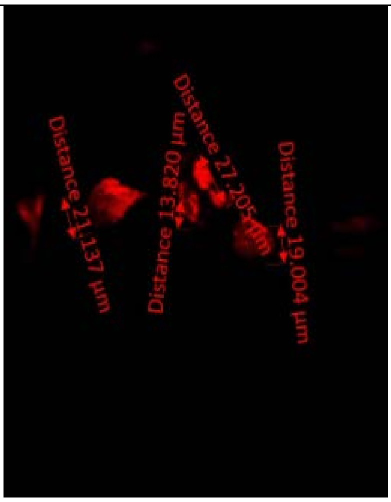	95% Ethanol	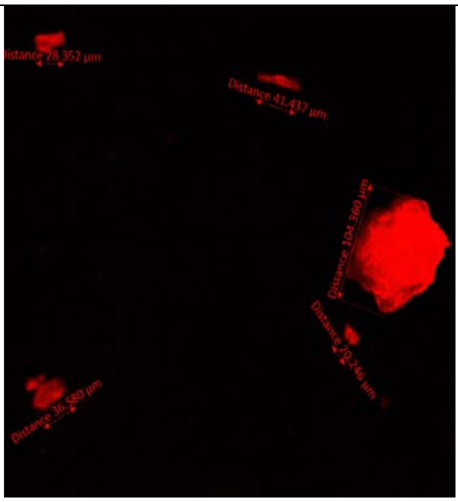
50% Ethanol	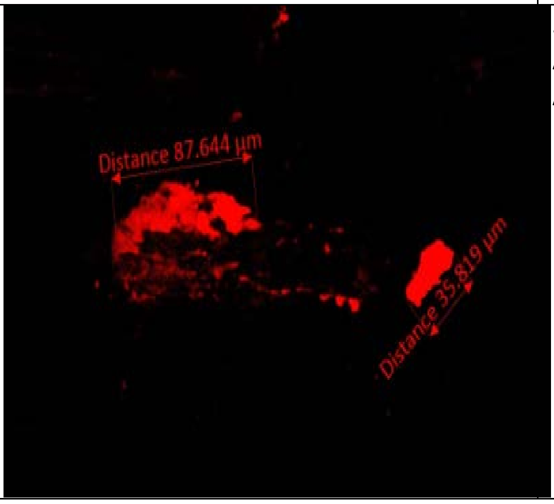	3% Acetic Acid	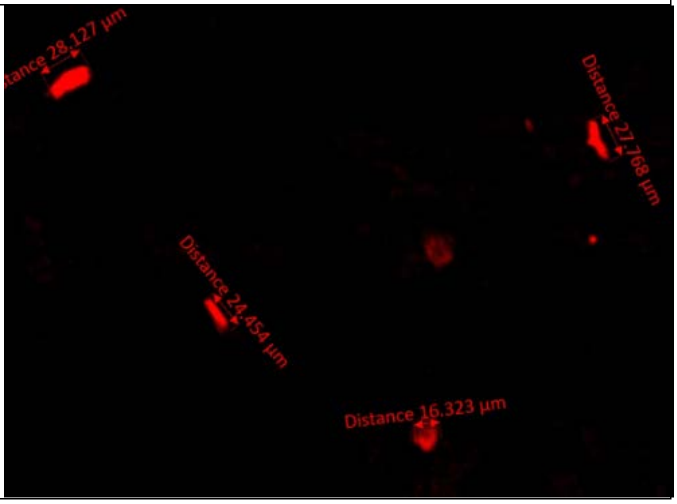

C: XPS polystyrene disposable plate at 70 °C.

<table>
<tr>
<td>10% Ethanol</td>
<td>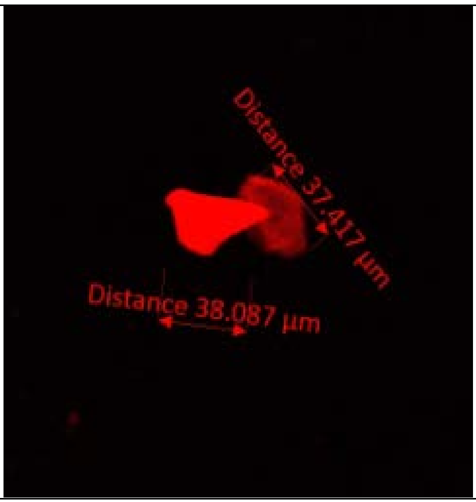
</td>
<td>95% Ethanol</td>
<td>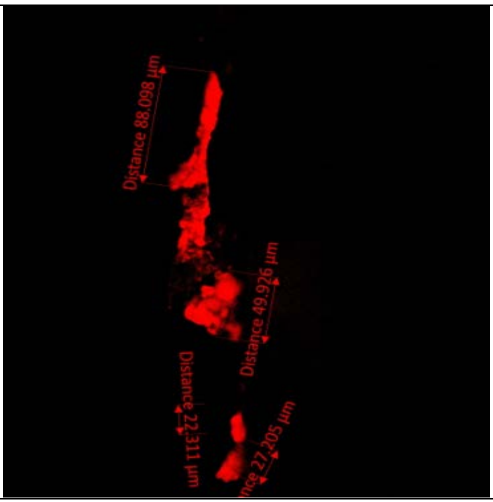
</td>
</tr>
<tr>
<td></td>
<td></td>
<td></td>
<td></td>
</tr>
<tr>
<td>50% Ethanol</td>
<td>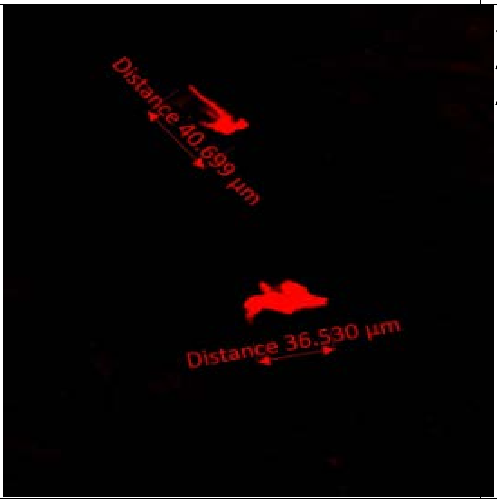
</td>
<td>3% Acetic Acid</td>
<td>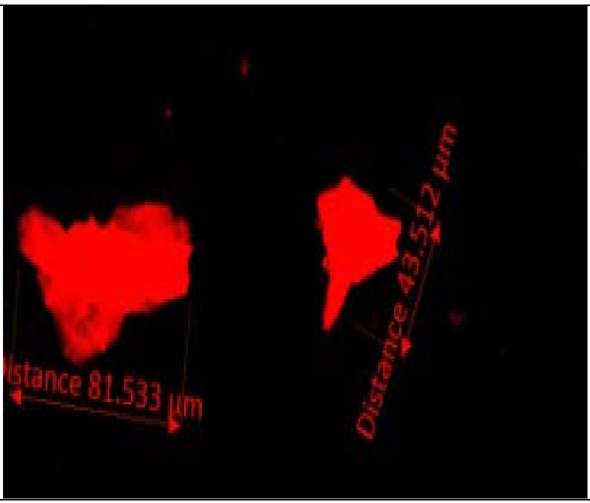
</td>
</tr>
</table>

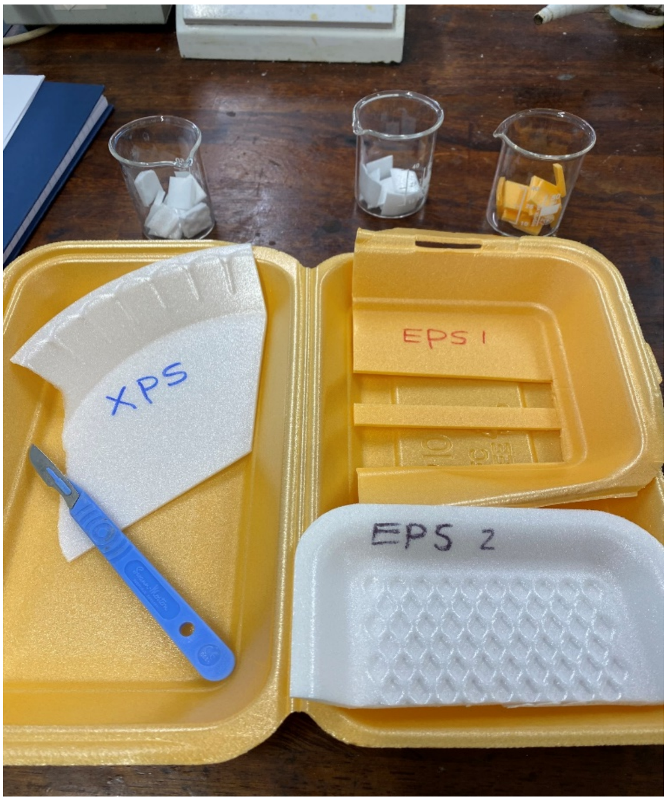Cutting process of samples	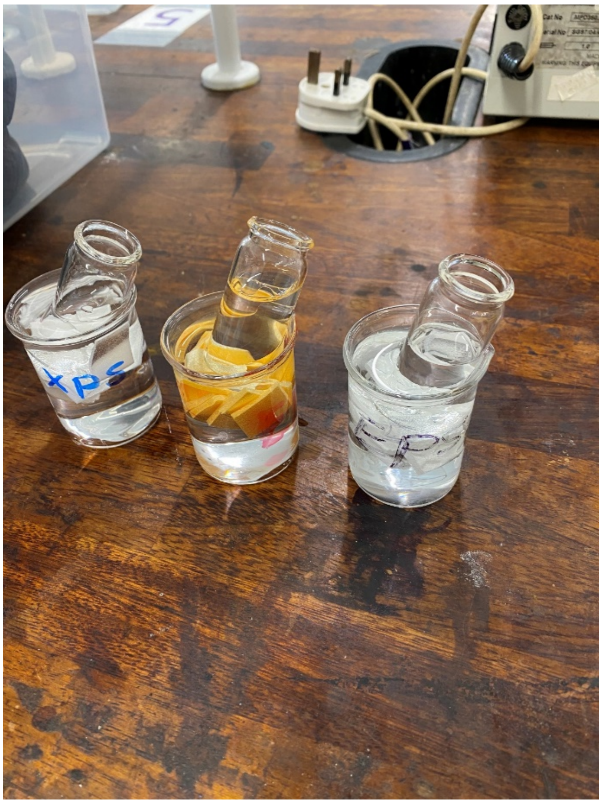Control experiment
Figure S1. Control experiment.	

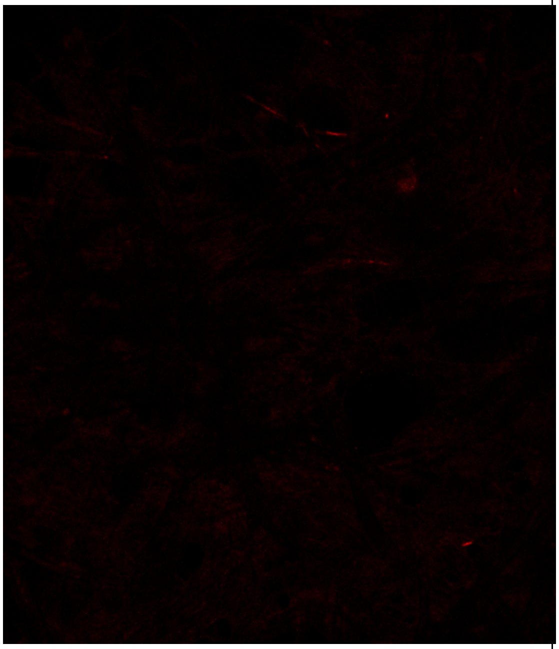Image of polycarbonate track-etch membranes with Nile Red stain	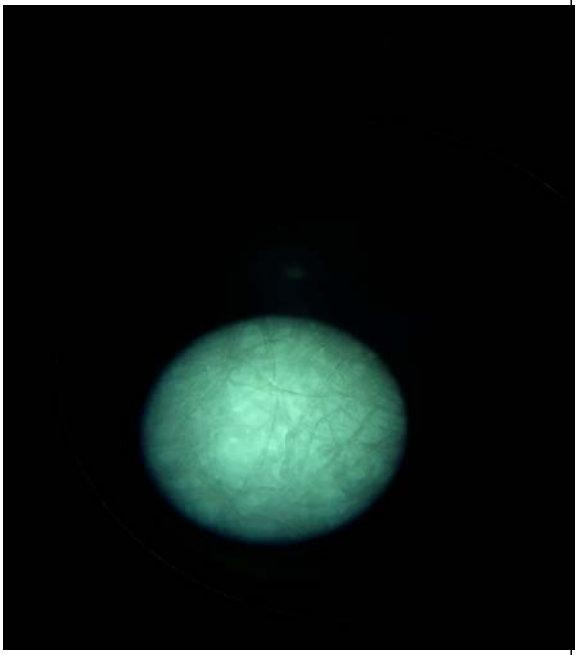Image of polycarbonate track-etch membranes without Nile Red stain

Figure S2. Images of clear of PCTE membrane from the microplastics-control experiment using Axio Observer Z1/7 microscope with an EC Plan Neofluar 10 × 0.30 m27 objective lens at an emission of 636 nm and an excitation of 559 nm; using an LSM800 MA Pmt2 imaging device.

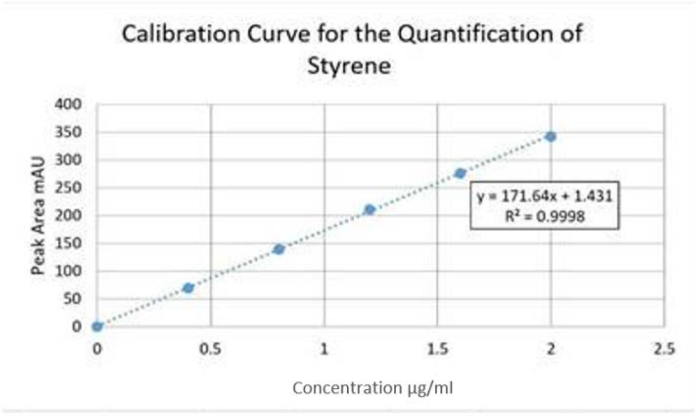

.

Figure S3. Calibration curve for styrene 0–2μg/mL.

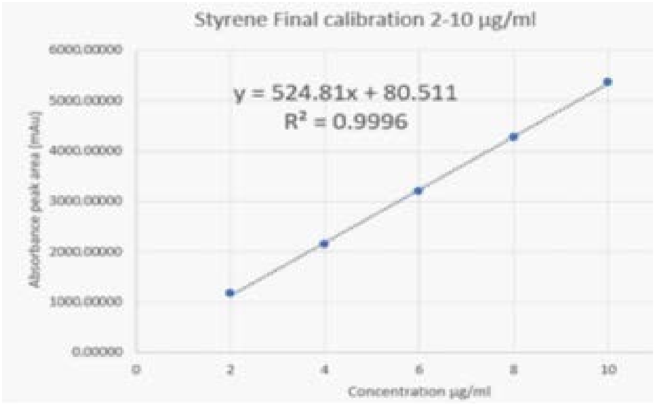

.

Figure S4. Calibration curve for styrene 2–10μg/mL.

Selective HPLC are presented below. The peak eluting in minutes being represented on the X-axis and the peak areas represented in *mAU* on the *Y-axis*).

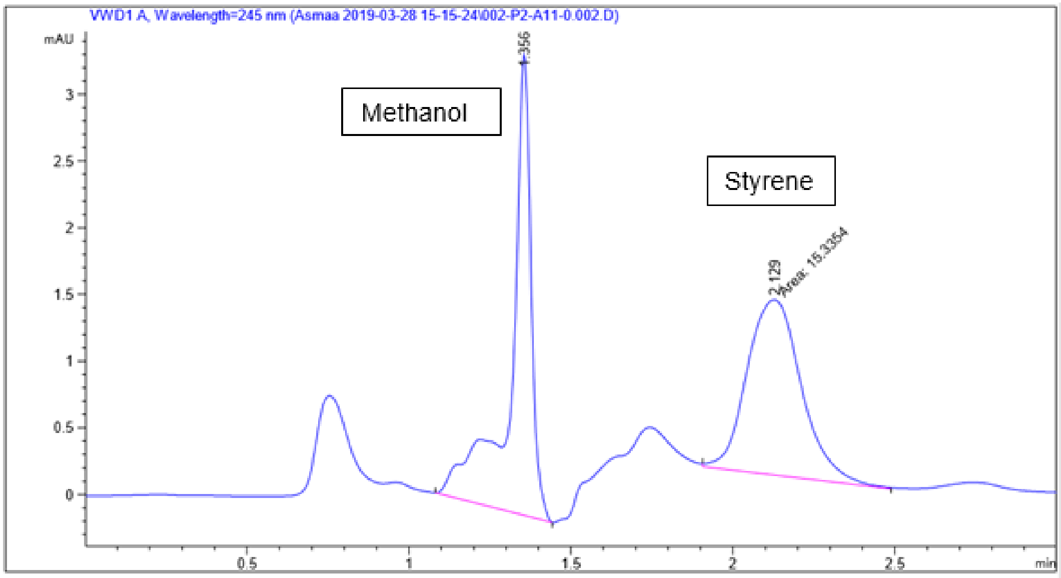

Figure S5. Chromatograph of a standard styrene solution with concentration 0.8 µg/mL.

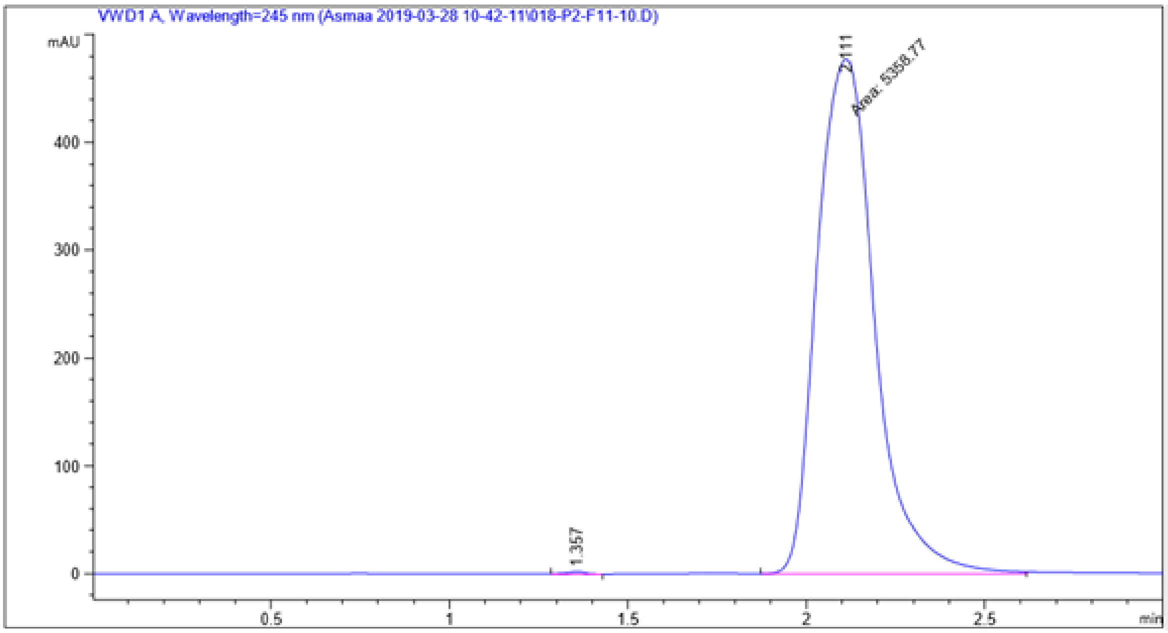

Figure S6. Chromatograph of a standard styrene solution with concentration 10 µg/mL.

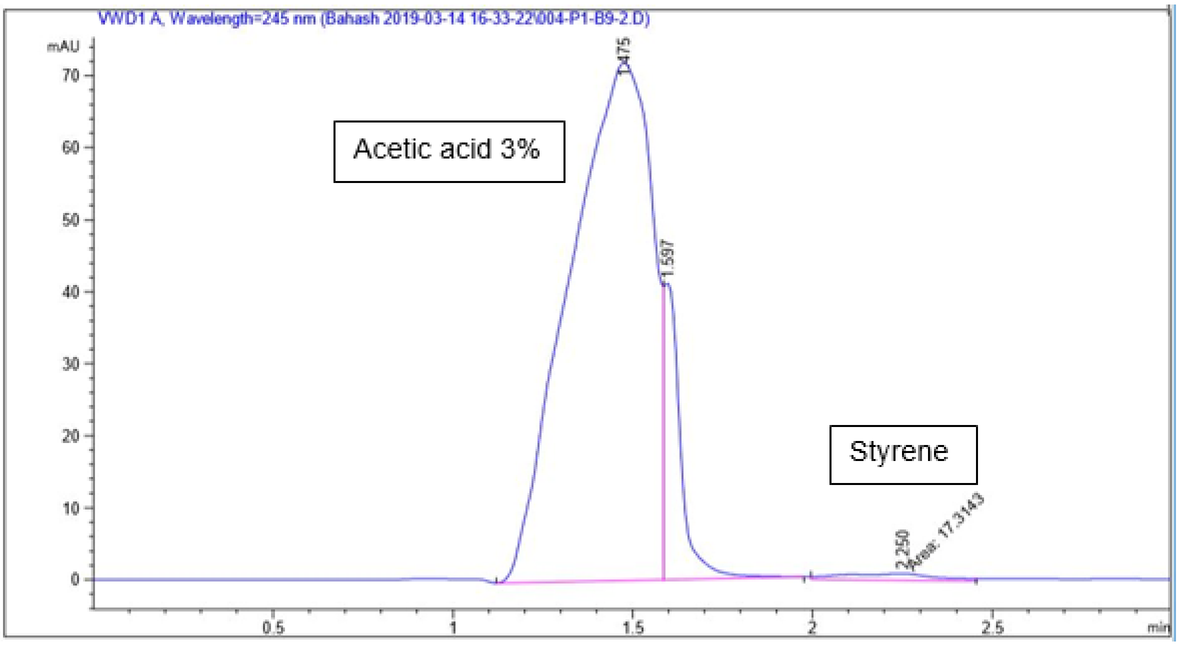

Figure S7**:** HPLC Migration of styrene in 3% acetic acid for 2 hours at 70 °C.

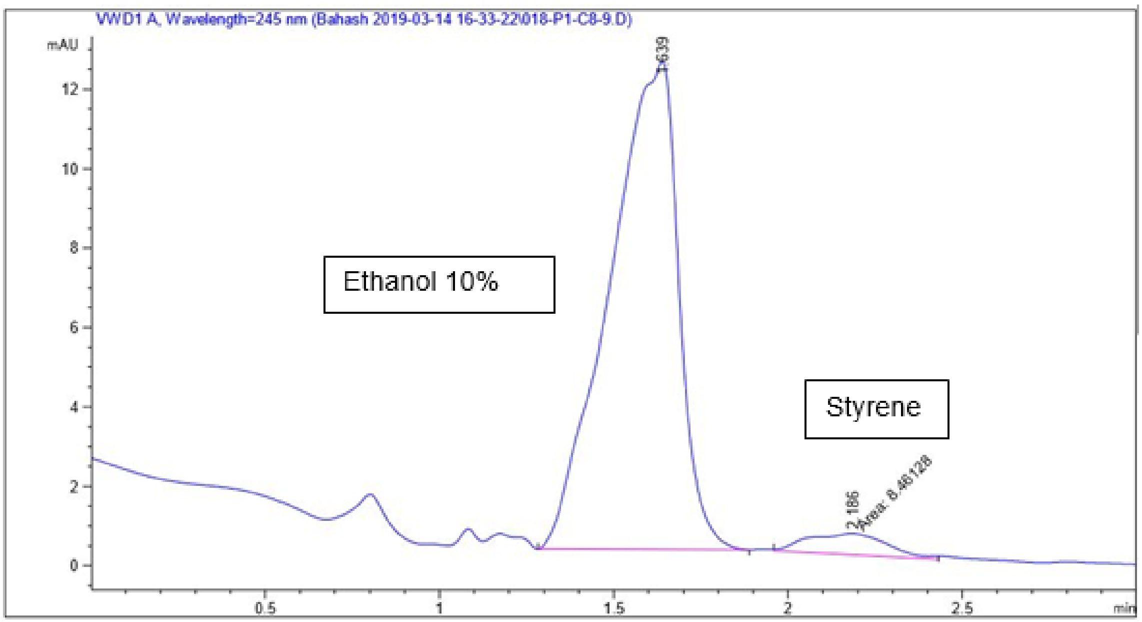

Figure S8**:** HPLC Migration of styrene in 10% ethanol for 240hours at 5 °C

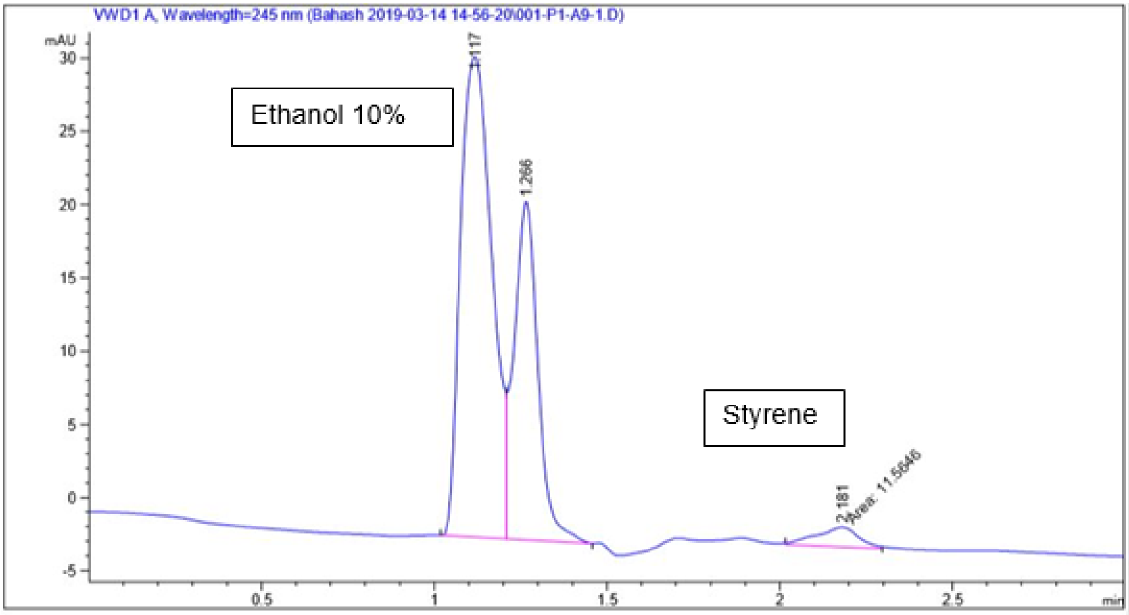

Figure S9**:** HPLC Migration of styrene in 10% ethanol for 2 hours at 70 °C.

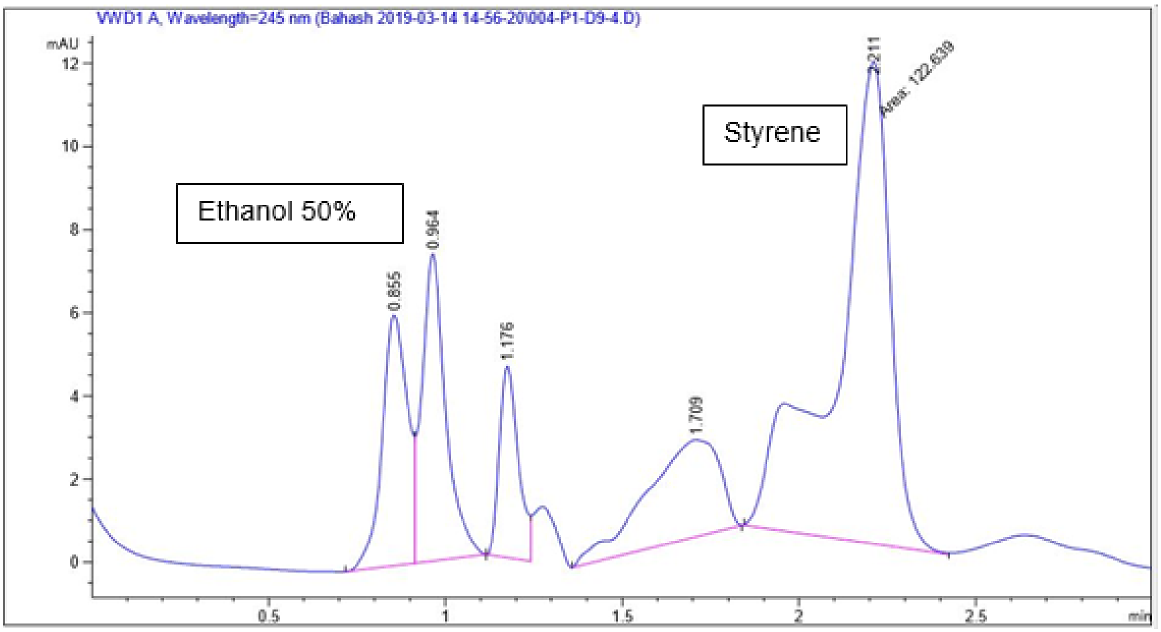

Figure S10**:** HPLC Migration of styrene in 50% ethanol for 2hours at 70 °C.

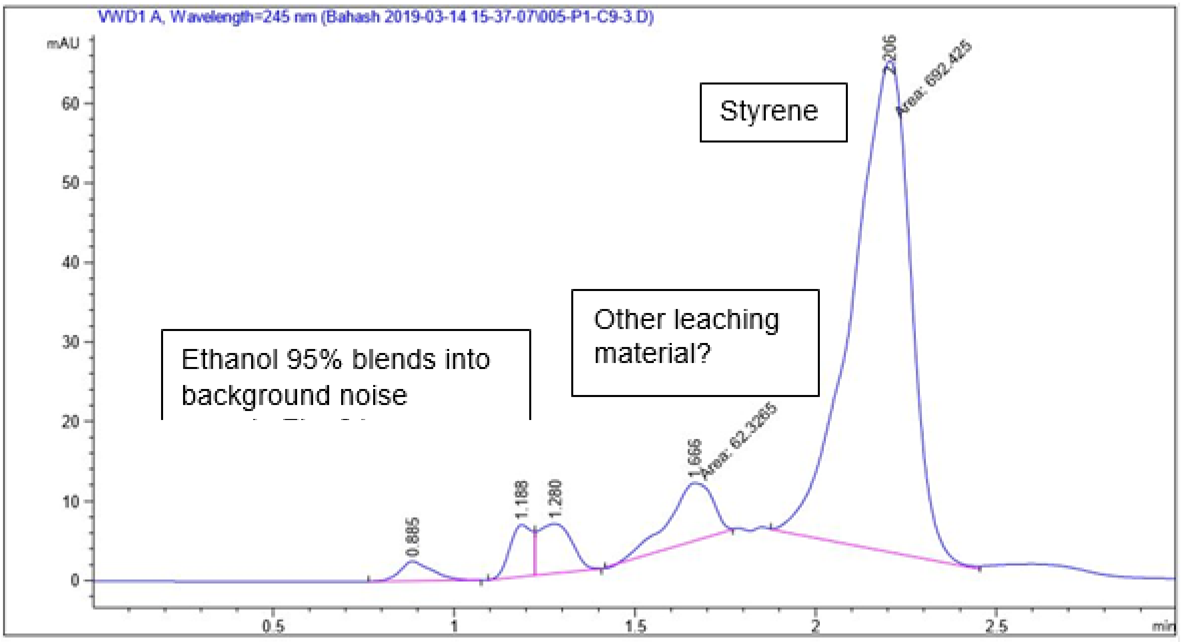

Figure S11: HPLC Migration of styrene in 95% ethanol for 2 hours at 70 °C.
